# The Amulet Device as Rescue Therapy in a Patient With Atrial Fibrillation and a Recurrent Gastrointestinal Bleed: A Case Report

**DOI:** 10.7759/cureus.99663

**Published:** 2025-12-19

**Authors:** Sarah I Zahid, Ayham Khan Ansari, Ans A Mahmood, Nadia I Zahid, Kifaya Tamimi

**Affiliations:** 1 Internal Medicine, Gulf Medical University, College of Medicine, Ajman, ARE; 2 Internal Medicine, Sheikh Shakhbout Medical City (SSMC), Abu Dhabi, ARE; 3 Medicine, Sheikh Shakhbout Medical City (SSMC), Abu Dhabi, ARE; 4 Family Medicine, Nassau University Medical Center, East Meadow, USA; 5 Internal Medicine, University of Sharjah, Sharjah, ARE

**Keywords:** amulet, gastrointestinal bleeding, nonvalvular atrial fibrillation, oral anticoagulation, watchman

## Abstract

In patients with non-valvular atrial fibrillation (AF) and a high stroke risk, long-term oral anticoagulation (OAC) is the standard of care. However, this becomes unfeasible in the presence of life-threatening bleeding, creating a significant therapeutic challenge. Percutaneous left atrial appendage occlusion (LAAO) offers a non-pharmacological alternative for stroke prevention. A 73-year-old female with persistent AF (CHA₂DS₂-VASc score of 6), end-stage renal disease on hemodialysis, and recurrent gastrointestinal (GI) bleeding was evaluated. Her GI workup suggested arteriovenous (AV) malformations of the small bowel. The patient's clinical course was complicated by recurrent hemorrhage despite a brief resumption of anticoagulant therapy, ultimately making long-term OAC an unsuitable strategy. She successfully underwent percutaneous LAAO with a 25 mm Amulet device (Abbott Medical, Chicago, IL, US). The procedure was uncomplicated, and she was discharged on a short course of dual antiplatelet therapy. This case demonstrates that the Amulet LAAO device is a safe and effective "rescue" strategy for stroke prophylaxis in patients with AF and an absolute contraindication to anticoagulation. It provides a definitive mechanical solution, resolving the critical conflict between thrombotic and hemorrhagic risks.

## Introduction

Atrial fibrillation (AF) is a leading cause of embolic stroke, and its management fundamentally relies on oral anticoagulation (OAC) to reduce this risk [1]. A significant clinical dilemma occurs when a patient's clear need for OAC is counterbalanced by a high risk of serious bleeding. Recurrent gastrointestinal (GI) hemorrhage is among the most prevalent reasons that patients cannot tolerate long-term anticoagulation [2].

In such complex scenarios, percutaneous left atrial appendage occlusion (LAAO) offers a guideline-recommended, mechanical alternative for stroke prevention [1]. This FDA-approved approach is physiologically rational, as the left atrial appendage is the origin of over 90% of intracardiac thrombi in non-valvular AF [1,3]. LAAO addresses the source of stroke without the systemic effects of anticoagulant drugs.

The Amulet device (Abbott Medical, Chicago, IL, US) represents an advanced iteration of LAAO technology, featuring a dual-seal design with an anchoring lobe and a separate occlusive disc [4]. This configuration promotes swift device endothelialization and provides immediate closure of the appendage. We describe a complex case of a patient with recurrent, life-threatening GI bleeding for whom the Amulet device constituted a pivotal and transformative rescue therapy.

## Case presentation

A 73-year-old female with a complex medical history, including persistent atrial fibrillation, end-stage renal disease (ESRD) managed with hemodialysis, diabetes mellitus, and severe pulmonary hypertension, was transferred for further management of recurrent melena and a significant hemoglobin drop below 7.0 g/dL, which necessitated a transfusion of packed red blood cells. Her gastrointestinal workup had revealed moderate erosive gastritis on esophagogastroduodenoscopy and blood-streaked stool in the cecum on colonoscopy, raising a strong suspicion for a small bowel bleeding source such as AV malformations. 

Following admission, the patient was hemodynamically stabilized. With no signs of active bleeding at that time, the gastroenterology team approved apixaban for stroke prevention, considering her elevated CHA₂DS₂-VASc score of 6 [1]. Her hospital course, however, was complicated by an episode of acute hypotension that required intensive care unit transfer and vasopressor support. A subsequent video capsule endoscopy indicated the presence of red blood in the stomach.

This pattern of recurrent gastrointestinal hemorrhage, in conjunction with her ESRD evident by her renal function tests (Table 1) and a significant fall risk secondary to diabetic peripheral neuropathy, led the multidisciplinary heart team to conclude that long-term anticoagulation posed a risk. The decision was therefore made to implement percutaneous LAAO as a definitive therapeutic measure.

**Table 1:** Pre-procedural renal function tests BUN: blood urea nitrogen; eGFR: estimated glomerular filtration rate; CKD-EPI: Chronic Kidney Disease Epidemiology Collaboration

Test	Patient’s Value	Reference Range
Creatinine	234 µmol/L	49-90 µmol/L
Urea Nitrogen (BUN)	9.4 mmol/L	2.5-7.1 mmol/L
eGFR (CKD-EPI)	19 mL/min/1.73m²	>60 mL/min/1.73m²

**Figure 1:**
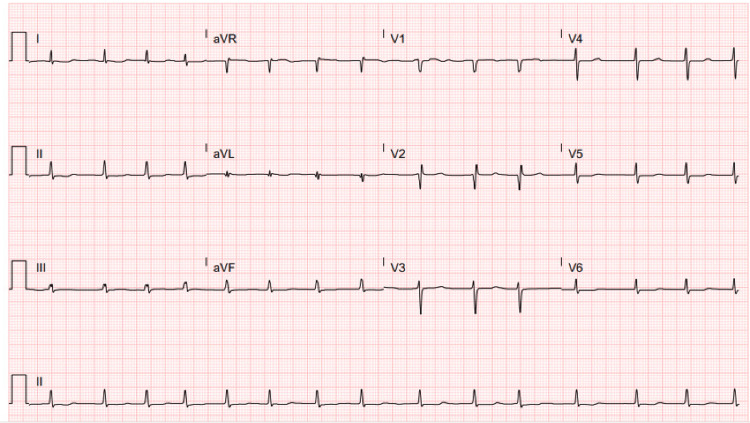
12-lead EKG tracing demonstrating atrial fibrillation

Initial diagnostic cardiac evaluation was performed using a 12-lead electrocardiogram (Figure 1). This tracing confirmed the presence of atrial fibrillation with a moderately controlled ventricular rate of 89 beats per minute. Additional analysis identified a prolonged QT interval, calculated at 674 milliseconds. The EKG also displayed specific morphological abnormalities indicative of a previous anteroseptal myocardial infarction, notably pathological Q waves and a notable loss of R-wave amplitude across the precordial leads V1 to V3.

A comprehensive hemodynamic assessment was obtained via right heart catheterization, providing critical data on her cardiopulmonary status (Table 2). The study revealed a significantly elevated mean pulmonary artery pressure of 44 mmHg. The pulmonary artery wedge pressure was measured at 19 mmHg, consistent with post-capillary pulmonary hypertension secondary to left heart pathology. Furthermore, the calculated pulmonary vascular resistance was elevated at 6 Wood Units, suggesting a mixed vascular pathophysiology involving both passive congestion and reactive pulmonary vasoconstriction. This hemodynamic profile confirmed the presence of advanced biventricular cardiac impairment.

**Table 2:** Hemodynamic Profile from Right Heart Catheterization

Parameter	Value	Normal Range
Right Ventricle (RV)	90/5/16 mmHg	15-30/2-8/3-12 mm Hg
Mean Pulmonary Artery Pressure (MPA)	44 mmHg	9-18 mm Hg
Pulmonary Artery Wedge Pressure (PAWP)	19 mmHg	6-12 mm Hg
Aortic Pressure	133/58/83 mmHg	100-140/60-90/70-105 mm Hg
Pulmonary Vascular Resistance (PVR)	6 Wood Units	0.5-1.5 Wood Units
Cardiac Output (CO)	4.9 L/min	4.0-8.0 L/min

Pre-procedural planning included cardiac computed tomography angiography, which visualized a windsock morphological variant of the left atrial appendage, deemed favorable for device implantation.

The implantation procedure was conducted under general anesthesia. Following an uncomplicated transseptal puncture, contrast angiography was performed to delineate the appendage anatomy. A 25 mm Amulet occluder device was subsequently advanced and deployed utilizing both fluoroscopic and transesophageal echocardiographic guidance. Device stability was verified using a standard tug test, and post-deployment imaging confirmed the absence of significant peri-device leakage. The procedure was completed without immediate complications.

Post-procedural pharmacological management consisted of dual antiplatelet therapy with aspirin and clopidogrel. Upon discharge, the therapeutic regimen included clopidogrel for one month and indefinite aspirin therapy. Subsequent clinical follow-up in three months confirmed the patient's continued clinical stability without the resumption of systemic anticoagulation.

## Discussion

This case highlights a complex clinical scenario in which LAAO emerged as the sole viable long-term approach for stroke prevention. The patient initially presented with life-threatening GI bleeding, secondary to small bowel AV malformations, leading to a temporary discontinuation of anticoagulation. Although apixaban was briefly reintroduced following clinical stabilization, evidence of recurrent gastrointestinal bleeding and her high-risk comorbidity profile solidified the consensus that long-term anticoagulation was not feasible. This situation emphasizes that the indication for LAAO includes not only patients with a single, absolute contraindication but also those with a recurrent or predictable pattern of major bleeding that precludes the safe use of OAC.

The presence of ESRD was a critical factor preventing long-term OAC. ESRD significantly elevates bleeding risk via two primary pathways. Firstly, it can lead to direct oral anticoagulants (DOACs) accumulation due to impaired renal excretion, resulting in supratherapeutic anticoagulant levels. Secondly, ESRD leads to an inherent bleeding tendency caused by uremia-induced platelet dysfunction [5]. In a patient with concurrent active or recurrent GI bleeding, these factors converge to create a prohibitively high risk of major hemorrhage, thereby establishing a mechanical alternative such as LAAO as the optimal management strategy.

Technical nuances: Amulet vs. Watchman

Device selection is a fundamental consideration in LAAO planning. Although both the Amulet and Watchman (Boston Scientific, Marlborough, MA, US) devices are commonly used, specific design differences guided the choice in this case. The Amulet device incorporates an advanced dual-seal design, consisting of a proximal disc and a distal lobe connected by a central waist. This structure is intended to provide both secure anchoring and comprehensive closure of the appendage. The disc, which is fabric-covered, is engineered to seal the left atrial appendage (LAA) ostium from the left atrial perspective, while the lobe provides deep anchorage within the LAA body [4,6,7].

Conversely, the Watchman FLX device utilizes a single, parachute-like frame that is implanted within the LAA body, depending on a compressive seal at its landing zone. A notable distinction lies in the fabric coverage. The Amulet's disc features fabric that extends to its periphery, a design aimed at reducing peri-device flow immediately after deployment. This is particularly advantageous in anatomies with a shallow landing zone or a wide ostium, as it allows for a more proximal seal [8]. The potential for a robust and immediate seal with the Amulet was a key feature that supported the decision to avoid post-procedural anticoagulation, which was a primary objective for our patient with active gastrointestinal hemorrhage.

Recent comparative trials, including the Amulet IDE study, have clarified these distinctions. While both devices showed high effectiveness in preventing ischemic stroke, the Amulet device demonstrated a significantly greater rate of complete LAA sealing, defined as the absence of peri-device leak greater than 5 mm, at both 45-day and 12-month follow-ups compared to the earlier Watchman 2.5 device [9]. Although the next-generation Watchman FLX has enhanced sealing properties, the fundamental design of the Amulet is intrinsically focused on achieving a result with minimal leak from the time of implantation. Additionally, the active fixation mechanism of the Amulet, which includes stabilizing wires, may provide superior stability in appendages that are highly contractile or have variable anatomy [10].

Clinical implications of the present case

The patient's overall condition, which included recurrent GI bleeding, ESRD, and a substantial risk of falls, necessitated a definitive mechanical solution that would allow for the permanent cessation of all anticoagulant medications. The design characteristics of the Amulet device were ideally suited to this objective. Its potential for prompt endothelial tissue growth and lower incidence of significant peri-device leak gave the clinical team confidence that the patient could be treated with only a short regimen of dual antiplatelet therapy, thereby directly mitigating the life-threatening risk of hemorrhage [9].

The objective data, from the EKG establishing the arrhythmic substrate to the right heart catheterization defining the severity of cardiopulmonary dysfunction, clearly outlined a high-risk patient for whom pharmacologic management was not viable. The successful implantation in a "windsock" morphology left atrial appendage, an anatomy known to be favorable for closure, further highlights the value of detailed pre-procedural imaging for achieving optimal results [11].

## Conclusions

Left atrial appendage occlusion with the Amulet device represents a therapeutic strategy for atrial fibrillation patients with absolute or recurrent contraindications to long-term anticoagulation. This case demonstrates its efficacy as a rescue therapy, successfully managing both stroke risk and hemorrhagic danger. The device's dual-seal technology provides mechanical advantages for achieving reliable LAA closure. A multidisciplinary heart team evaluation is essential for identifying appropriate candidates and ensuring optimal outcomes with this intervention, which resolves the critical conflict between thrombosis and hemorrhage prevention.
